# Molecular chaperones at the host–virus interface: heat shock protein roles in HIV-1 and emerging insights for HIV-2 and dual infection

**DOI:** 10.3389/fcimb.2025.1729538

**Published:** 2025-12-03

**Authors:** Sabastine Eugene Arthur, Kirk Klogo, Emmanuel Kobina Mensah, Maame Ama Pentsiwa Cudjoe, Adomia Baaba Mensah, Nyamekye Amoabeng Ankrah, Catherine Laaripuoh Omosule, Evelyn Yayra Bonney, George Boateng Kyei

**Affiliations:** 1 Medical and Scientific Research Centre, University of Ghana Medical Centre, Legon, Accra, Ghana; 2 Noguchi Memorial Institute for Medical Research, University of Ghana, Legon, Accra, Ghana; 3 Department of Pathology and Immunology, Washington University School of Medicine in St. Louis, St. Louis, MO, United States; 4 Departments of Medicine and Molecular Microbiology, Washington University School of Medicine in St. Louis, St. Louis, MO, United States; 5 Departments of Molecular Microbiology, Washington University School of Medicine in St. Louis, St. Louis, MO, United States

**Keywords:** heat shock proteins, HIV latency, HIV-1 and HIV-2, HSF1, latency-reversing agents (LRAs)

## Abstract

Heat shock proteins (HSPs) are essential molecular chaperones involved in protein folding, cellular stress responses, and homeostasis. Recent studies reveal their critical and dual roles in the human immunodeficiency virus (HIV) life cycle, both promoting and restricting viral replication, latency, and immune modulation. This review synthesizes current evidence on how key HSP families, HSP70, HSP90, and HSP40, interact with HIV proteins such as Tat, Rev, Nef, and Vpx, affecting viral transcription, protein trafficking, and latency. We also highlight Heat Shock Factor 1 (HSF1) as a direct regulator of the HIV-1 long terminal repeat (LTR), facilitating latency reversal via recruitment of transcriptional co-activators like p300 and P-TEFb. In HIV-2, distinct HSP-mediated mechanisms, such as HSP40-facilitated nuclear import of Vpx-associated pre-integration complexes, may contribute to its lower replication rates and deeper latency. The review further discusses the incorporation of HSPs into virions, their potential as therapeutic targets, including HSP90 inhibitors and HSF1 modulators, and identifies gaps in understanding HSP roles in HIV-2 and dual infections. We propose future research directions that could harness host stress-response machinery to address HIV persistence and latency.

## 1 Introduction

### 1.1 Global impact of HIV and rationale for host–directed cure targets

Despite dramatic ART-mediated reductions in AIDS-related mortality, HIV persists because proviral DNA integrates into long-lived cell populations and establishes transcriptional latency. In 2024, an estimated 40.8 million people were living with HIV, with approximately 65% of cases in the WHO African Region, and 630,000 AIDS-related deaths were reported globally (Global HIV & AIDS statistics, 2025). This latent reservoir cannot be cleared by ART and represents the primary barrier to a cure. Consequently, cure research has increasingly focused on host biological systems that govern latency, transcriptional reactivation, cell survival, and stress adaptation, rather than exclusively targeting viral enzymes. Among these, cellular stress response pathways, including heat shock proteins (HSPs), have emerged as critical modulators of proviral transcriptional competence and reservoir stability (Nastiti et al., 2024).

### 1.2 HIV-1, HIV-2, and dual infection: mechanistic implications for HSP biology

HIV-1 is globally dominant, highly replication-competent, and exhibits transcriptionally active LTRs responsive to NF-κB and NFAT signaling. In contrast, HIV-2 is less transmissible, maintains lower steady-state viremia, and often exhibits deeper baseline latency. In regions of West Africa, dual HIV-1/HIV-2 infections have been reported, though literature on HSP involvement in HIV-2 and dual infection remains limited. These biological distinctions are mechanistically important because viral exploitation of chaperone networks, co-factor dependency, and stress response wiring may differ across HIV types. Preliminary evidence suggests Vpx-mediated interactions with HSP40 (DNAJB6) in HIV-2 may constitute a differential latency architecture not present in HIV-1, highlighting the need for comparative evaluation of HSP-mediated regulation (Kerkau et al., 1997; Berger and Cimarelli, 2014; Nastiti et al., 2024).

### 1.3 Cure strategy frames: where heat shock biology fits

HIV cure strategies include sterilizing (eradication) cures, functional cures, and hybrid approaches combining reservoir reduction with immune-mediated clearance (Cillo and Mellors, 2016). Sterilizing cures aim to eliminate all replication-competent HIV. Prominent examples include the Berlin, London, and Düsseldorf patients, who achieved ART-free remission after hematopoietic stem cell transplantation from CCR5Δ32 homozygous donors (Gupta et al., 2020; Jensen et al., 2023); the New York patient, who remained off ART for over 14 months after a dual umbilical cord and adult stem cell transplant from a CCR5Δ32 (Hsu et al., 2023); and the Geneva patient, demonstrating prolonged remission via host-directed mechanisms beyond CCR5Δ32 editing (Sáez-Cirión et al., 2024).

Functional cures seek durable viral control without elimination, often via CRISPR-mediated modification of viral or host genes (Ophinni et al., 2018; Gao et al., 2020; Nguyen et al., 2021; Fan et al., 2022). The “Mississippi baby” exemplifies this, maintaining an undetectable viral load after early ART initiation before eventual rebound (Persaud et al., 2013).

Hybrid approaches combine reservoir reduction with immune boosting. “Shock and kill” uses latency-reversing agents (LRAs) to expose latent viruses, while “block and lock” enforces deep latency (Kula-Pacurar et al., 2021). Heat shock proteins (HSPs) and HSF1 now emerge as central regulators of proviral silencing versus activation, with HSP40 and related pathways representing promising, underexplored targets for host-directed cure strategies (Persaud et al., 2013; Berger and Cimarelli, 2014; Brown, 2015; Cillo and Mellors, 2016; Ophinni et al., 2018; Brown, 2020; Gao et al., 2020; Kula-Pacurar et al., 2021; Nguyen et al., 2021; Fan et al., 2022; First case of HIV cure in a woman after stem cell transplantation reported at CROI-2022, 2022; Nastiti et al., 2024; Tioka et al., 2025).

## 2 Heat shock proteins: overview and biological functions

Heat shock proteins (HSPs) are an evolutionarily conserved family of molecular chaperones essential for maintaining cellular proteostasis, particularly under conditions of physiological or environmental stress. Their expression is upregulated in response to stimuli such as heat, oxidative stress, infection, heavy metals, and other protein-destabilizing events (Udaka et al., 1993; Hartl, 1996). Despite being initially characterized in the context of heat shock responses, it is now well established that HSPs play constitutive roles in protein folding, trafficking, refolding of misfolded proteins, prevention of protein aggregation, and the regulation of apoptosis (Hartl et al., 2011; Finka and Goloubinoff, 2013).

In humans, HSPs are categorized based on molecular weight into families such as small HSPs (sHSPs or HSPB), HSP40 (DNAJ), HSP60 (chaperonins), HSP70 (HSPA), HSP90 (HSPC), and HSP110 (HSPH), among others (**Table 1**) (Csermely et al., 1998; Kampinga et al., 2009). Importantly, these families are now formally defined according to shared evolutionary homology and the presence of characteristic, highly conserved functional domains, consistent with current nomenclature standards (Kampinga et al., 2009). Each family consists of multiple isoforms, many of which exhibit tissue-specific expression and subcellular localization, and can be found in the cytosol, mitochondria, endoplasmic reticulum, and nucleus. These proteins do not function in isolation, but often operate as part of multi-protein complexes, cooperating with co-chaperones or other cellular machinery to mediate their activities. For instance, the ATPase activity and protein-folding capacity of HSP70 is heavily influenced by HSP40 (Kampinga and Craig, 2010).

**Table 1:** Heat shock proteins with roles in HIV life cycles.

HSP type	Alternative name	Number of isoforms	Molecular weight (kDa)	Intracellular locations	Extracellular presence	Biological cellular roles	In-Text references
Small HSP (sHSP)	HSPB	~10	12–42/43	Nucleoli, nucleoplasm, Golgi apparatus, cytosol	No	Protein stability, folding, stress response	(Hartl et al., 2011; Hu et al., 2022)
HSP40	DNAJ	~49	~40	Nucleoli, vesicles, mitochondria, microtubules, cytosol, plasma membrane	Some studies suggest extracellular presence in eukaryotes	Co-chaperone assisting HSP70 in protein folding	(Fan et al., 2003; Kampinga and Craig, 2010; Iyer et al., 2021)
HSP60	Chaperonins/HSPD	2 subfamilies (HSPD1, CCT1–CCT8)	~58	Mitochondria, nucleoplasm, cytosol, cytoskeleton, plasma membrane	Yes	Facilitates protein folding using ATP hydrolysis, forms cylindrical structure	(Ishida et al., 2018; Iyer et al., 2021)
HSP70	HSPA	~13 (HSPA1–HSPA14, excluding HSPA10)	~70	Nucleoplasm, vesicles, mitochondria, ER, Golgi apparatus, microsomes, cytosol	No	Assists protein folding, refolding, translocation, regulates apoptosis	(Jäättelä et al., 1998; Nollen and Morimoto, 2002; Finka and Goloubinoff, 2013)
HSP90	HSPC	~5 (HSP90AA1, HSP90AA2, HSP90B1, HSP90AB1, TRAP1)	~90	Cytosol, mitochondria (TRAP1), ER (HSP90B1)	No	Interacts with kinases, steroid hormone receptors, regulates protein stability	(Stechmann and Cavalier-Smith, 2004; Chen and Ott, 2020)
HSP110	HSPH	~4 (HSPH1–HSPH4)	Variable	ER, cytosol, nucleoplasm, centrosome	No	Assists in heat shock response, maintains protein integrity	(Subjeck et al., 1982; Iyer et al., 2021).

In viral infections, including those caused by retroviruses like HIV, HSPs are key players. They are implicated in processes that facilitate or restrict viral replication, latency, and immune evasion. Several lines of evidence suggest that HSPs are deeply implicated in the HIV replication cycle and its latency control. For example, in lymphocytes from HIV-infected individuals, HSP70 is overexpressed (Agnew et al., 2003). HSP40 family members have also been shown to interact directly with HIV accessory proteins such as Nef, modulating viral transcription (Kumar and Mitra, 2005). Some HSP40 isoforms like DNAJB1 facilitate the nuclear translocation of Nef, while others such as DNAJB6 modulate interactions with HIV-2’s Vpx protein, influencing trafficking of the pre-integration complex (PIC) (Cheng et al., 2008; Kumar et al., 2011). Further, HSP60 and HSP70 have been detected within HIV virions, raising questions about their potential roles in viral assembly or infectivity (Gurer et al., 2002). Also, HSP90, a master regulator of the latent reservoir, promotes the folding of proteins required for transcriptional reactivation of latent HIV by influencing key host signaling pathways such as NF-κB, NFAT, and AP-1 (Noorsaeed et al., 2025).

Given these diverse yet highly specific interactions, HSPs have garnered significant interest as potential therapeutic targets in HIV cure research. Their ability to modulate the activation state of latently infected cells, either by promoting or repressing viral gene expression, positions them as central components in “shock and kill” or “block and lock” strategies. Moreover, Heat Shock Factor 1 (HSF1), a transcriptional regulator of HSP genes, has been shown to bind the HIV-1 long terminal repeat (LTR) and contribute to viral reactivation, further supporting the role of heat shock responses in latency reversal (Pan et al., 2016). Therefore, understanding the nuanced roles of HSPs in HIV-1 and HIV-2 infections is crucial for informing future therapeutic development.

### 2.1 Roles of heat shock proteins in viral infections, immunomodulation, and latency

Heat shock proteins (HSPs) are not only vital for cellular homeostasis but also play multifaceted roles during viral infections. They interact with both host and viral components, making them central to the viral life cycle, influencing viral entry, replication, assembly, immune evasion, and latency regulation in both DNA and RNA viruses (Lubkowska et al., 2021).

HSPs play a dual role in viral infections. They can promote immune recognition and host protection by facilitating viral antigen presentation via MHC molecules (Li et al., 2002; Furuta and Eguchi, 2020). They, particularly, the extracellular HSPs can also, act as danger signals, activating dendritic cells and stimulating cytokine production through toll-like receptors (TLRs), particularly TLR2 and TLR4 (Hu et al., 2022). Conversely, viruses can subvert HSP functions to inhibit host immunity, to disrupt antigen processing, prevent apoptosis of infected cells and block proteasomal degradation (Iyer et al., 2021). Thus, HSPs may have both virus-supportive and host-protective roles in viral infections, making them attractive targets for antiviral strategies, particularly for complex viruses like HIV, which establish lifelong latency and evade immune clearance.

Furthermore, HSP may have roles in HIV latency. Chaudhary et al. (2016) in 2016 found that HSP70-binding protein 1 (HSPBP1), a co-chaperone of HSP70, is highly expressed during HIV latency and inhibits reactivation of HIV through the NF-κB pathway. Downregulation of HSPBP1 during reactivation highlights its possible role in maintaining latency. Similarly in 2023, Iyer et al. (2023) discovered that the HIV Tat protein suppresses HSPBP1 expression by interfering with its promoter, further emphasizing the intricate regulation of HSPs during the viral life cycle.

#### 2.2.1 HSP70

HSP70 is one of the most extensively studied chaperone systems in virology and plays crucial roles in viral protein folding, genome replication, capsid assembly, and intracellular trafficking (Niewiarowska et al., 1992; Gurer et al., 2002; Burch and Weller, 2004; Seo et al., 2018). In DNA viruses such as adenovirus, HSP70 promotes capsid disassembly and nuclear trafficking (Niewiarowska et al., 1992), while in hepatitis B virus (HBV) it enhances capsid assembly and RNA encapsulation (Seo et al., 2018), and in herpes simplex virus (HSV) it facilitates nuclear sequestration and gene expression (Burch and Weller, 2004). For some RNA viruses, including enteroviruses and dengue virus (Picornaviridae and Flaviviridae families, respectively), HSP70 is required for efficient replication and translation of viral RNA (Gurer et al., 2002). HSP70 is often incorporated into viral replication complexes and, in certain cases, into virions, supporting its role as a viral cofactor (Gurer et al., 2002).

In HIV-1, HSP70 exerts both proviral and antiviral effects depending on the infection context. On the proviral side, HSP70 facilitates folding, stabilization, and intracellular trafficking of viral proteins such as Gag and Gag-Pol precursors, supporting virion assembly and infectivity (Udaka et al., 1993; Hartl, 1996). Conversely, HSP70 stabilizes host restriction factors such as APOBEC3G, thereby inhibiting Vif-mediated degradation and reducing virion infectivity and replication (Agnew et al., 2003; Sugiyama et al., 2011; Sugiyama et al., 2013). Pharmacological induction of HSP70 further confirms its ability to block Vif-mediated APOBEC3G degradation, while knockdown of cellular HSP70 enhances Vif function, promoting APOBEC3G degradation and viral replication (Sugiyama et al., 2011).

Importantly, HSP70 activity is often modulated by co-chaperones such as DNAJB8 (Hsp40/DNAJ family), which assist HSP70 in recognizing, stabilizing, or targeting specific client proteins. In the context of HIV-1, DNAJB8 promotes autophagic-lysosomal degradation of Vif, indirectly enhancing HSP70’s stabilization of APOBEC3G and further reducing viral infectivity (Chand et al., 2023). This illustrates that HSP70’s antiviral effects are not isolated but operate within a broader co-chaperone network, adding mechanistic nuance to its regulation of the Vif–APOBEC3G axis.

Recent studies indicate that HSP70 family isoforms are not functionally interchangeable and exert differential effects depending on cellular stress, metabolic state, and infection phase (Iyer et al., 2021). This isoform-level heterogeneity helps explain the simultaneous proviral and antiviral roles of HSP70 in HIV-1. Systematic analyses across *in vitro*, ex vivo, and limited *in vivo* models suggest that these bidirectional effects are consistent biological phenomena rather than experimental artifacts (Iyer et al., 2021; Nastiti et al., 2024). Taken together, HSP70 and its co-chaperones act as central molecular determinants of HIV proteostasis, virion infectivity, and latency permissiveness.

#### 2.2.2 HSP90

HSP90 is another highly conserved chaperone with broad roles across viral pathogens, including HIV, influenza virus, HCV, HSV, emerging coronaviruses and multiple oncogenic DNA viruses (Csermely et al., 1998; Kampinga et al., 2009; Kampinga and Craig, 2010). In HIV-1 biology, HSP90 supports viral protein stability, regulates signal transduction pathways required for efficient replication, and contributes to virion infectivity. HSP90 also stabilizes key host transcriptional regulators such as NF-κB and the P-TEFb complex, both of which are central drivers of LTR activation and latency reversal (Agnew et al., 2003; Kumar and Mitra, 2005). Importantly, HSP90 has been identified within HIV virions themselves, suggesting a direct proviral incorporation role during assembly and infectivity (Cheng et al., 2008).

Recent mechanistic work reinforces that HSP90 is not simply a generic stress protein, but rather a specific host co-factor necessary for efficient HIV transcriptional reactivation from latency, and this process appears to be HSP90-dependent (Lubkowska et al., 2021). *In vivo* evidence further demonstrates that HSP90 inhibition prevents viral rebound following latency reversal attempts, indicating that HSP90 inhibitors function to block latency disruption rather than activate it (Joshi et al., 2016).

#### 2.2.3 HSP40

HSP40 (DNAJ family) functions primarily as a co-chaperone for HSP70, regulating substrate specificity, folding kinetics, and client handoff dynamics (Gurer et al., 2002; Kumar et al., 2011). Beyond HIV, HSP40 isoforms have been implicated in HSV, dengue virus, influenza virus, hepatitis viruses, and Ebola, where they influence viral protein translation, nuclear import, and immune evasion (Pan et al., 2016). In HIV-1, specific HSP40 family members interact with viral proteins, including Nef, modulating transcriptional competence and contributing to both immune signaling perturbation and replication efficiency (Lubkowska et al., 2021; Chen et al., 2022). HSP40 also influences PIC handling and integration, particularly within resting T cells.

Of clinical and comparative relevance to this review, HIV-2 biology appears to be more dependent on HSP40-linked pathways than HIV-1 in key early replication steps, as Vpx has been shown to interact with DNAJB6 to support nuclear import of the PIC, providing a plausible molecular basis for differential latency architecture between HIV-1 vs HIV-2 (Nastiti et al., 2024). Recent isoform-level mapping also suggests that individual HSP40 family proteins exert non-redundant, and in some cases opposing, effects on HIV-1 replication and transcriptional activation, supporting deeper isoform mechanistic exploration (Iyer et al., 2021).

#### 2.2.4 HSP60

HSP60 is a mitochondrial chaperonin that supports protein folding, mitochondrial proteostasis and apoptosis regulation, and has been linked to multiple viral infections including HBV, HCV, HSV, HPV, influenza viruses and several respiratory viruses (Li et al., 2002; Furuta and Eguchi, 2020; Hu et al., 2022). In the context of HIV-1, HSP60 has been detected within virions and may contribute to capsid stability, virion maturation, and post-entry fitness (Iyer et al., 2021). HSP60 also intersects with mitochondrial stress signaling pathways, which is relevant because mitochondria play a central role in metabolic fitness, inflammasome signaling, and survival of long-lived reservoir cells (Chaudhary et al., 2016).

Emerging literature synthesizing HSP–HIV correlations supports that HSP60 involvement may be broader than initially appreciated, including regulatory influence on permissive stress states and immunometabolic adaptations in infected cells (Nastiti et al., 2024). Meanwhile, broader viral comparative work suggests HSP60 functions as a conserved node implicated in host response modulation across multiple virus families (Lubkowska et al., 2021). Future studies should prioritize HSP60 isoform-level roles in HIV reservoir cells to determine whether targeted modulation of mitochondrial chaperone stress programs can meaningfully influence latency stability and reservoir durability.

## 3 Comparative perspectives: HSP interactions in HIV-1 vs. HIV-2 and HIV-1/2 dual infection

### 3.1 Known differences in HSP regulation between HIV-1 and HIV-2

HIV-1 and HIV-2 differ not only in clinical outcomes and geographic prevalence but also in how they interface with host molecular chaperone systems. HIV-1 extensively utilizes HSP70, HSP90, and HSP40 families to fold viral proteins, stabilize replication complexes, and support transcriptional activation through interactions with Tat, Rev, Nef, and Vif to promote viral gene expression and replication (Agnew et al., 2003; Kumar and Mitra, 2005; Ko et al., 2019). Mechanistically, these patterns are consistent with broader HSP pro-viral roles seen in multiple viral systems (Lubkowska et al., 2021) and HIV-1 isoform-specific function mapping (Iyer et al., 2021).

In contrast, HIV-2 mechanistic chaperone data remain limited, but emerging evidence demonstrates differential architecture. HIV-2 uses the accessory protein Vpx to antagonize SAMHD1, and this function is facilitated by interaction with the HSP40 isoform DNAJB6, which enhances nuclear import of the HIV-2 pre-integration complex in non-dividing cells (Cheng et al., 2008). HIV-2’s promoter structure contains fewer NF-κB binding sites, potentially rendering it less responsive to stress-induced transcriptional programs mediated by HSF1 and HSP90, contributing to deeper basal latency states (Nastiti et al., 2024). These divergent patterns of HSP chaperone utilization may therefore contribute to the slower progression and distinct reservoir phenotype seen in HIV-2.

### 3.2 Potential mechanistic insights in individuals living with HIV-1/2 (dual infections)

HIV-1/2 dual infections occur predominantly in West Africa, where both viral types co-circulate. Clinical studies suggest viral interference, sequential dominance, and context-dependent shifts over time, with HIV-1 often eventually becoming the predominant replicating strain in the absence of ART (van der Loeff et al., 2006; Campbell-Yesufu and Gandhi, 2011). HIV-1 demonstrates stronger reliance on HSP90, HSP70, and HSF1 as reactivation scaffolds, aligning with its greater transcriptional responsiveness to inflammatory signaling (Iyer et al., 2021; Lubkowska et al., 2021). In contrast, HIV-2 may use an alternative chaperone axis via HSP40 isoforms such as DNAJB6 to support Vpx-mediated PIC trafficking (Cheng et al., 2008), resulting in reduced reliance on canonical HSP90/HSF1-dependent reactivation circuits.

Whether these differential HSP dependency profiles translate into selective reactivation or functional competition between viral types *in vivo* is not yet known. At present, there are no direct comparative functional studies examining HSP isoform competition or HSP-mediated selective latency reversal in dually infected individuals. This remains a mechanistically plausible (yet untested) hypothesis that warrants directed investigation.

These considerations have implications for the design of therapeutic strategies. For example, HSP90/HSF1-driving LRAs may theoretically show differential efficacy against HIV-1 versus HIV-2; however, this has not been experimentally demonstrated and requires explicit testing in dual-infection systems.

A concise comparative summary of HSP utilization across HIV-1, HIV-2 and HIV-1/2 dual infection contexts is provided in **Table 2**.

**Table 2:** Comparative summary of HSP interactions in HIV-1, HIV-2, and HIV-1/2 dual infection.

Viral type context	Dominant HSP interactions (examples)	Implications	Representative evidence base
HIV-1	HSP70/HSP90/HSP40 support folding, replication complex stability and transcriptional activation (Tat/Rev/Nef/Vif)	HSP90/HSF1 pathway strongly linked to latency reversal responsiveness	(Timmons et al., 2020; Iyer et al., 2021; Lubkowska et al., 2021)
HIV-2	Alternative chaperone architecture with Vpx–SAMHD1 antagonism and suggested HSP40 involvement (DNAJB6)	Lower NF-κB dependence, may contribute to deeper basal latency states	(Nastiti et al., 2024)
HIV-1/2 dual infection	Mechanistic HSP isoform competition is plausible based on differential viral HSP reliance, but has not been demonstrated empirically in dual-infected primary cells or clinical cohorts	Differential HSP reliance raises the testable hypothesis that LRAs acting through HSP90/HSF1 could preferentially affect HIV-1 over HIV-2; however this remains untested and requires directed comparative ex vivo studies in dual infection	(no direct studies)

### 3.3 Gaps in knowledge and opportunities for study

As summarized in **Table 2**, current evidence for differential HSP utilization between HIV-1, HIV-2, and HIV-1/2 dual infection is uneven, with almost all characterized mechanistic HSP studies derived from HIV-1, and no direct dual-infection HSP comparative studies available. Despite growing recognition of HSP involvement in HIV biology, significant gaps remain in understanding how these interactions differ between HIV-1, HIV-2, and dual infections. The bulk of published data focuses on HIV-1 (Joshi et al., 2013; Mercier et al., 2013; Urano et al., 2013; Anderson et al., 2014; Xia et al., 2015; Chaudhary et al., 2016; Joshi et al., 2016; Mbonye et al., 2018; Priyanka et al., 2020; Chand et al., 2021; Chand et al., 2023; Iyer et al., 2023; Nastiti et al., 2024), leaving HIV-2 vastly understudied. Only a few HSP isoforms, such as DNAJB6, have been linked to HIV-2-specific processes. Comprehensive profiling of HSP expression and binding partners in HIV-2-infected cells is lacking.

Key areas where knowledge is limited include the mechanisms by which HSPs influence viral replication, latency, stress responses, and virion composition in HIV-2 and dual infections. In particular, the roles of specific HSP isoforms in HIV-2 infection remain largely undefined, and it is unclear whether HSP-mediated processes differ fundamentally from those in HIV-1. Moreover, no studies have examined HSP signatures in individuals dually infected with HIV-1 and HIV-2, nor how HSP-targeting latency-reversing strategies perform in these contexts. Proteomic and transcriptomic studies of latent reservoirs from such patients could illuminate type-specific stress responses and chaperone utilization.

Bridging these knowledge gaps is essential for developing pan-HIV cure strategies. Future research should employ high-resolution techniques, such as single-cell transcriptomics, isoform-specific knockdowns, and CRISPR-Cas9 editing, to delineate the precise roles of HSPs in HIV-2 and HIV-1/2 infections. Integrative approaches that combine virology, molecular chaperone biology, and latency modelling will be crucial for translating these insights into effective therapeutic interventions (Joshi et al., 2013; Urano et al., 2013; Anderson et al., 2014; Xia et al., 2015; Mbonye et al., 2018; Chand et al., 2021; Iyer et al., 2023; Nastiti et al., 2024).

## 4 Heat shock pathway modulation and HIV latency control strategies

### 4.1 Heat shock factor 1 and HIV latency: implications for “shock and kill” strategies

**Figure 1:**
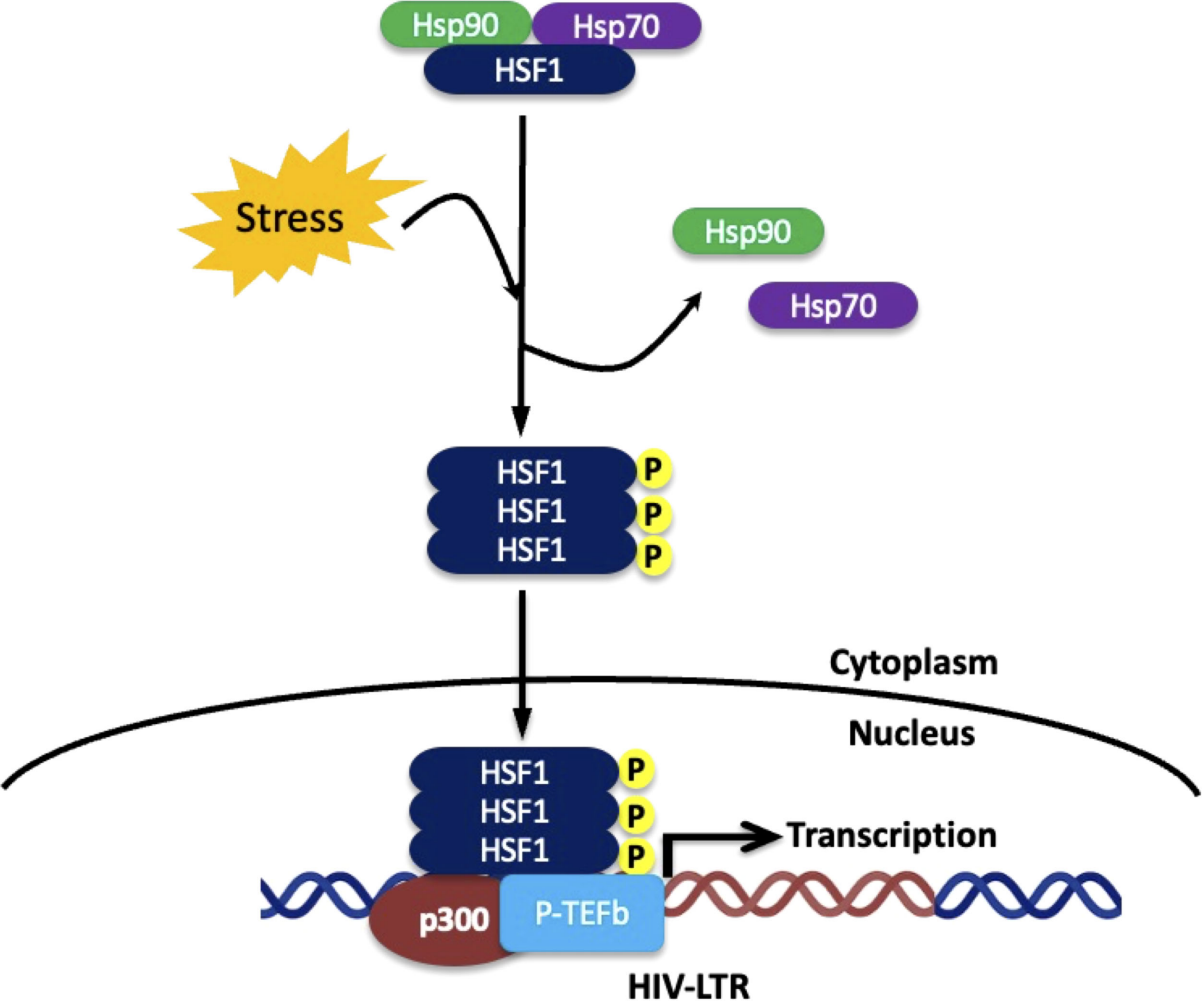
Canonical activation of HSF1 during cellular stress and its role in HIV latency reversal. Under non-stress conditions, HSF1 remains monomeric and inactive in the cytoplasm, inhibited by HSP70 and HSP90. During proteotoxic or thermal stress, chaperones are diverted to misfolded proteins, releasing HSF1 to trimerize and become phosphorylated, forming an active transcriptional complex. Activated HSF1 trimers translocate to chromatin, bind heat shock elements, and recruit co-activators such as p300 and P-TEFb to promote transcriptional elongation. This same mechanism enables HSF1 to bind the HIV LTR and recruit p300 and P-TEFb, driving latency reversal.

Heat Shock Factor 1 (HSF1) is the master transcriptional regulator of the heat shock response. Under non-stress conditions, HSF1 remains monomeric and inactive in the cytoplasm, bound by molecular chaperones such as HSP70 and HSP90. Upon exposure to stress, including heat, oxidative stress, or viral infection, HSF1 undergoes trimerization, phosphorylation, and translocation to the nucleus. Once in the nucleus, it binds to heat shock elements (HSEs) within the promoter regions of heat shock protein genes, driving their expression (Åkerfelt et al., 2010; Hartl et al., 2011)​. More recent systematic interrogation confirms stress-induced HSF1 activation as a conserved trigger for chaperone upregulation in human T-cell systems (Nastiti et al., 2024). **Figure 1** depicts the canonical HSF1 activation pathway and its role in driving transcription from the HIV LTR.

Recent findings have expanded the role of HSF1 beyond regulating chaperone genes, identifying its involvement in viral gene regulation, most notably in HIV. Pan and colleagues have provided compelling evidence that HSF1 binds directly to a conserved region within the HIV-1 LTR (Pan et al., 2016), leading to the recruitment of co-activators such as the histone acetyltransferase p300 and the P-TEFb complex, which is composed of CDK9 and cyclin T1. Together, they initiate and elongate transcription from the LTR, effectively reactivating latent virus. More recent data further reinforce that HSF1 activity is a decisive upstream determinant of LRA potency, where inhibition of HSF1 reduces latency reversal induced by several LRA classes *in vitro* and ex vivo (Timmons et al., 2020).

Additionally, HSF1 is upregulated in latently infected T cells subjected to heat stress (Pan et al., 2016). It is one of the most differentially expressed and functionally enriched transcriptional regulators during latency reversal. The knockdown of HSF1 significantly diminishes the reactivation of latent HIV, whereas HSF1 overexpression alone is sufficient to induce LTR activity, even in the absence of external inducers (Iyer et al., 2021; Xu et al., 2021). Complementarily, new mechanistic work expands pathway integration of HSF1 with cellular stress buffering that connects HSF1-regulated chromatin remodeling to inducible reservoir destabilization (Nastiti et al., 2024).

The exact binding motif of HSF1 within the LTR remains under investigation, but its close proximity to known NF-κB and Sp1 binding sites suggests a collaborative regulatory model. HSF1 may act synergistically with other transcription factors that are commonly involved in LTR activation, such as NFAT and AP-1 (Pan et al., 2016). These interactions suggest a broader stress-sensing module embedded within the LTR that responds to environmental or intracellular cues by triggering viral reactivation. Most of the research has focused on HIV-1, and it is imperative to determine if HSF1 binds similarly to the HIV-2 LTR.

The ability of HSF1 to bind the HIV-1 LTR and induce transcriptional reactivation has significant implications for HIV cure research, particularly within the context of the “shock and kill” strategy. This approach aims to purge the latent reservoir by pharmacologically or physiologically inducing proviral transcription “shock”, followed by immune- or drug-mediated clearance of reactivated cells “kill” (Margolis et al., 2016).

HSF1-mediated reactivation is unique, and physiologically relevant for the “shock” phase. Heat stress, febrile illness, or pharmacologic activators of HSF1 can increase its activity, triggering the transcription of latent HIV. Pan and colleagues have demonstrated that mild heat stress could induce reactivation in latent HIV models via HSF1, pointing to stress-induced reactivation as a viable therapeutic strategy (Pan et al., 2016). Based on this converging evidence, HSF1 is increasingly recognized as a therapeutically actionable stress-responsive transcriptional axis that can be leveraged for latency reversal, rather than simply a secondary consequence of stress biology.

Importantly, HSF1 appears to coordinate other latency-reversing pathways, serving as a multifaceted activator also integrates horizontally with other stress-responsive transcription modules by promoting chromatin remodeling through p300 recruitment and stimulating transcriptional elongation via P-TEFb., HSF1. These mechanisms align closely with current pharmacological approaches employing histone deacetylase inhibitors (HDACis), BET bromodomain inhibitors, and protein kinase C agonists. Some of these compounds may act in part by indirectly modulating HSF1 activity or its upstream signaling cascades (Zelin and Freeman, 2015). This aligns with current cure-directed LRAs that incorporate HSF1 axis contribution into potency predictions and failure modes (Tioka et al., 2025).

Nevertheless, the use of HSF1 as a therapeutic target presents several challenges. Systemic activation of HSF1 may upregulate other stress-response genes, potentially leading to off-target effects or unwanted immune activation. Furthermore, as HSF1 controls the expression of numerous cytoprotective proteins, including HSP70 and HSP90, indiscriminate activation may promote cell survival, thereby reducing the efficacy of the “kill” phase.

To circumvent these concerns, future approaches may involve the use of targeted HSF1 modulators or LTR-specific mimetics that activate transcription without triggering a complete stress response. Alternatively, heat shock mimetics or localized hyperthermia could be investigated as physical tools to induce site-specific HSF1 activation. Recent translational forward-looking analyses specifically recommend precision biophysical modulation over global pharmacologic activation (Tioka et al., 2025).

In HIV-2 and dual infections, where latency is more deeply entrenched and transcriptional activity is lower (Saleh et al., 2017; Bruggemans et al., 2023), HSF1-based strategies may face additional hurdles. It is unclear whether HIV-2 LTRs are as responsive to HSF1 activation, and whether other viral or host regulatory factors override HSF1-driven transcription in these contexts. Identifying and comparing stress response pathways in HIV-1 vs. HIV-2-infected cells will be key to adapting HSP-1 in the shock and kill approach for broader use.

### 4.2 HSP-centered “block and lock”: isoform-specific latency stabilization mechanisms

Heat shock proteins also contribute to latency maintenance. Several HSP isoforms can suppress viral transcription, restrict essential viral protein stability, or reinforce antiviral host factors, rather than promote reactivation. For example, recent evidence confirms that isoform-selective HSP70 interactors can down-modulate NF-κB-driven proviral transcriptional activity in primary T cell models under physiologic stress states (Nastiti et al., 2024). Isoform-specific HSP40 family members, including DNAJB subclades, can reinforce host restriction factor efficiency and promote selective degradation of viral accessory proteins (Iyer et al., 2021). These isoform-specific suppressive effects provide a mechanistic basis for HSP-centered “block and lock” strategies in which selective reinforcement of protective chaperone subnetworks would stabilize latency.

HSP70 and HSP40 families are diverse and exhibit substantial functional redundancy. Different isoforms regulate distinct viral steps, and their effects vary across cell types, metabolic states, and reservoir compartments (Iyer et al., 2021). A recent systematic review of HSP–HIV interactions highlights that isoform patterns differ across reservoir phenotypes, suggesting that therapeutic latency stabilization could be targeted without inducing a global stress program (Nastiti et al., 2024). This contrasts with broad activation of the heat shock response, which risks inducing HSF1-dependent transcriptional rebound.

Pharmacologic data supports this model. Inhibition of HSF1 reduces the efficacy of multiple latency-reversing agents *in vitro* and ex vivo (Timmons et al., 2020). This indicates that selective dampening of stress transcription factor signaling could stabilize latency, particularly in inflammatory microenvironments where subthreshold activation cues persist. Forward-looking translational cure frameworks propose pairing isoform-targeted HSP modulatory strategies with immune-state-aligned containment to enforce reservoir suppression post-ART (Tioka et al., 2025). Future therapeutic development may therefore integrate isoform-specific HSP70/HSP40 enhancers with immune surveillance optimization and post-ART viral containment strategies (Tioka et al., 2025).

## 5 Therapeutic implications and future directions

The expanding understanding of heat shock proteins (HSPs) as pivotal regulators of HIV replication, latency, and immune interactions has unlocked new possibilities for therapeutic intervention in HIV cure research. These molecular chaperones, alongside their key regulator, Heat Shock Factor 1 (HSF1), are intricately involved in pathways governing viral gene expression, immune activation, and cellular stress responses. Notably, HSPs can exhibit both proviral and antiviral effects, making them complex therapeutic targets that require precise, context-specific modulation.

To provide a clear and comprehensive overview of existing strategies targeting HSPs in HIV cure research, we have summarized these approaches in **Table 3**. This table details the targeted HSPs, their mechanisms of action, therapeutic applications, benefits, and potential challenges. Such a synthesis underscores the dual nature of HSP modulation, either promoting viral latency or driving reactivation, thereby informing the design of future therapeutic strategies.

**Table 3:** Summary of therapeutic strategies targeting HSPs in HIV cure research.

Strategy	Targeted HSP(s)	Mechanism of action	Therapeutic application	Advantages	Challenges	References
HSP90 inhibition	HSP90AA1, HSP90AB1	Disrupts stabilization of NF-κB, P-TEFb, and Tat; impairs transcriptional reactivation	Latency reversal; virion destabilization	Effective reactivation in some models; broad impact on host co-factors	Toxicity, off-target effects, compensatory stress pathways	(Pan et al., 2016; Noorsaeed et al., 2025)
HSP70 inhibition	HSPA1A, HSPA1B	Modulates APOBEC3G–Vif; overexpression protects APOBEC3G, knockdown enhances Vif function.	May reduce virion infectivity via APOBEC3G; can sensitize latent reservoirs (context-dependent)	Impairs essential proviral processes	Dual antiviral and pro-viral roles; ubiquitous expression	(Agnew et al., 2003; Sugiyama et al., 2011)
HSPBP1 modulation	HSP70 co-chaperone	Downregulation leads to NF-κB activation and HIV reactivation	Latency reversal	Selectively enriched in latent cells	Requires further validation *in vivo*	(Chaudhary et al., 2016)
HSF1 activation	Transcription factor regulating HSPs	Binds HIV-1 LTR; recruits P-TEFb and p300; reactivates latent virus	Latency-reversing agent (LRA)	Mimics physiological stress-induced reactivation	May cause widespread activation of stress genes	(Pan et al., 2016; Chand et al., 2021)
HSP-triggered immune activation	HSP60, extracellular HSP70	Activates TLRs and innate immunity; promotes cytokine release	Immune clearance after latency reversal (“kill” phase)	Enhances immune visibility of reactivated cells	Potential for off-target inflammation or immune suppression	(Flohé et al., 2003; Zanin-Zhorov and Cohen, 2007)
Combinatorial strategies	Multiple HSP isoforms	Combine LRA, immune activation, and apoptosis sensitization	Broad-spectrum latency management and clearance	Synergistic targeting of multiple latency checkpoints	Complexity, toxicity, and patient-specific tuning required	(Bartz et al., 1994; Gurer et al., 2002; Kumar et al., 2011)

### 5.1 HSP inhibitors and HIV-1 latency

Previous studies have shown that heat shock proteins are intimately involved in controlling HIV-1 transcriptional activity and latency states. HSP70 family modulators can suppress HIV-1 replication through inhibition of NF-κB-mediated activation of viral gene expression, reinforcing transcriptional silencing (Chaudhary et al., 2016). HSP90 is also deeply involved in HIV latency biology. Rather than functioning as direct LRAs, HSP90 supports the host transcriptional machinery required for efficient LTR activation, and therefore HSP90 inhibition blocks reactivation rather than promotes it (Anderson et al., 2014). Multiple independent studies have demonstrated that HSP90 activity is permissive for latency reversal, whereas HSP90 inhibition prevents viral rebound following LRA exposure (Anderson et al., 2014; Joshi et al., 2016; Janssens et al., 2024; Noorsaeed et al., 2025). Moreover, because HSP90 signaling intersects directly with the HSF1 stress transcriptional axis, inhibition of HSF1 has been shown to attenuate the magnitude of latency reversal across diverse LRAs (Timmons et al., 2020). Taken together, these data reposition HSP90 (and upstream stress regulators such as HSF1) as host co-factors required for efficient latency reactivation, thereby aligning HSP90/HSF1 pharmacologic inhibition more closely with “block and lock” concepts rather than conventional “shock and kill” conceptualization.

### 5.2 HSP modulation for HIV reservoir reduction

Beyond latency reversal, modulating HSP activity could contribute to reducing the size or persistence of the HIV reservoir. For instance, suppression of HSPs that promote the survival of infected cells, such as HSP70 and HSP90, may sensitize latent reservoirs to immune clearance or apoptosis following reactivation (Rodina et al., 2007). Moreover, combining HSP modulation with immune-based approaches such as therapeutic vaccines, broadly neutralizing antibodies (bNAbs), or cytotoxic T lymphocyte (CTL) enhancement could improve the “kill” phase of “the shock and kill” strategy. Further, HSPs such as HSP60 and extracellular HSP70 are known to function as danger signals, activating innate immune receptors like TLR4 and stimulating cytokine production (Davies et al., 2006). This immunostimulatory property could be harnessed to enhance immune recognition of reactivated HIV-infected cells.

Recent findings demonstrate that HSP-mediated effects in HIV latency biology are bidirectional and not exclusively aligned with shock-and-kill paradigms. HSP70 family members can enforce latency via NF-κB suppression (Chaudhary et al., 2016), while HSP90 is required to maintain transcriptional competency necessary for reactivation, such that HSP90 inhibition blocks rather than induces latency reversal (Anderson et al., 2014; Joshi et al., 2016; Noorsaeed et al., 2025). These positions selected HSP modulators also within a “block and lock” conceptual space—where silencing is stabilized rather than reversed, suggesting that isoform-selective HSP targeting could serve both reservoir reduction and latency maintenance therapeutic objectives depending on context.

However, this approach has challenges as immune cell function impairment or induction of compensatory stress responses that support viral persistence may occur. Therefore, combinatorial regimens that include isoform-selective HSP modulators and immune-enhancing therapies may offer the best balance between efficacy and safety (Davies et al., 2006; Rodina et al., 2007; Anderson et al., 2014; Haase and Fitze, 2015; Chaudhary et al., 2016).

### 5.3 Challenges and considerations for targeting host proteins

Targeting host proteins like HSPs introduces several inherent complexities. Unlike direct-acting antivirals that target viral enzymes, host-directed therapies risk off-target effects and cellular toxicity. Many HSPs are ubiquitously expressed and essential for basic cell functions, especially under stress. Long-term or systemic inhibition of HSP90, for example, may lead to hepatotoxicity, gastrointestinal symptoms, or suppression of other essential signaling pathways (Okamoto et al., 2008).

Furthermore, HSPs play dual roles in HIV biology, supporting both viral replication and host defenses. For instance, HSP70 can stabilize apolipoprotein B mRNA editing enzyme, catalytic polypeptide 3G (APOBEC3G), a host restriction factor, while also assisting in viral protein folding (Sugiyama et al., 2011). Similarly, HSP40 isoforms show isoform-specific activity: some promote transcription, while others suppress it (Ko et al., 2019). This functional duality makes selective targeting, rather than broad inhibition, a critical goal for future drug development.

Another layer of complexity lies in HIV subtype diversity and dual HIV-1 and HIV-2 infection contexts. As already discussed, because HIV-2 and HIV-1/2 dual infections may engage HSPs differently, cure strategies targeting HSP pathways may need to be type-optimized.

### 5.4 Emerging tools and technologies

To advance this field, novel tools and models are required to unravel the precise roles of HSPs in different stages of HIV infection. These include:

- CRISPR-based genetic editing which allows isoform-specific knockout or modulation of HSP genes in latent cell models.
- Proteomics and interactomics, enabling the mapping of HSP-viral protein interactions at high resolution across different HIV subtypes and latency states.
- Single-cell transcriptomics, which can characterize stress-response profiles in latently infected cells versus productively infected or uninfected cells.
- Latency models incorporating patient-derived cells, especially from individuals with HIV-2 or dual infections, to validate the relevance of *in vitro* findings.

Furthermore, drug screening platforms using HSP–HIV interaction assays could help identify small molecules that either disrupt proviral HSP functions or enhance antiviral HSP activities. These compounds could form the basis of next-generation LRAs, latency stabilizers, or virion disruptors.

HSPs and their regulatory networks are rich, underexplored targets for HIV cure strategies. From latency reversal to reservoir sensitization and immune activation, they occupy multiple strategic nodes in host-virus interaction. With careful modulation and targeted delivery, they may help bridge the gap between current antiretroviral therapy and functional or sterilizing cures. However, realizing this potential requires addressing the biological complexity and therapeutic challenges of targeting host proteins, particularly in diverse viral subtypes and dual infection scenarios.

It is important to interpret HSP-centered therapeutic hypotheses within the limitations of current experimental systems. Many mechanistic data sets on HSP–HIV interactions are derived from transformed T-cell lines or acute infection models, which may not fully recapitulate primary reservoir biology (Siliciano and Greene, 2011). Reservoir composition also varies across tissue compartments, age, sex, genetic background, and HIV subtype, factors that may differentially shape HSP pathway dependency. Targeting host stress systems carries distinct safety considerations because HSPs regulate essential cellular proteostasis across multiple physiological processes (Haase and Fitze, 2015). Therefore, translation of chaperone-directed interventions will require isoform-specific pharmacology, primary cell validation, and integrated immunometabolic modeling to avoid broad stress toxicity while exploiting latency control potential (Joshi et al., 2016).

## 6 Conclusion

Heat shock proteins occupy central regulatory positions at the interface of HIV replication, latency, stress biology, and host cell survival. Evidence to date demonstrates that HSP70, HSP90, HSP40, HSP60 and HSF1 do not operate as unidirectional proviral factors, but instead participate in bidirectional control systems capable of either facilitating viral reactivation or reinforcing transcriptional silencing depending on cellular context, environmental stress inputs, and isoform engagement. Comparative differences between HIV-1 and HIV-2 suggest that chaperone dependencies may be type-specific, with emerging evidence indicating distinct Vpx–HSP40 axis utilization in HIV-2 and potential competitive chaperone resource allocation in dual infection states. Moving forward, future cure-directed efforts should prioritize isoform-selective modulation, primary cell and tissue-based validation, and integrated immunometabolic modeling to delineate therapeutic windows. As HSP systems represent a high-value host-directed axis, resolving their dual antiviral/proviral roles will be critical for informing rational chaperone-targeting strategies across both shock-and-kill and block-and-lock paradigms (Cheng et al., 2008).
